# Stakeholder opinions on perceived sub-standard emergency obstetric and newborn care in Ghana

**DOI:** 10.1186/s12913-024-10936-x

**Published:** 2024-04-12

**Authors:** Alice Ayawine, Mathias J. A. Asaarik, Roger A. Atinga

**Affiliations:** 1 School of Public Health, Catholic University of Ghana, Fiapre, Sunyani P.O. Box 363, Ghana; 2 School of Nursing and Midwifery, Catholic University of Ghana, Fiapre, Sunyani P.O. Box 363, Ghana; 3 Department of Health Services Management, University of Ghana Business School, Legon, Accra Ghana

**Keywords:** EmONC, Opinions, Ghana, Sub-standard, Stakeholder

## Abstract

**Background:**

Sub-Saharan Africa is unlikely to achieve sustainable development goal (SDG) 3 on maternal and neonatal health due to perceived sub-standard maternal and newborn care in the region. This paper sought to explore the opinions of stakeholders on intricacies dictating sub-standard emergency obstetric and newborn care (EmONC) in health facilities in Northern Ghana.

**Methods:**

Drawing from a qualitative study design, data were obtained from six focus group discussions (FGDs) among 42 health care providers and 27 in-depth interviews with management members, clients and care takers duly guided by the principle of data saturation. Participants were purposively selected from basic and comprehensive level facilities. Data analysis followed Braun and Clarke’s qualitative thematic analysis procedure.

**Results:**

Four themes and 13 sub-themes emerged as root drivers to sub-standard care. Specfically, the findings highlight centralisation of EmONC, inadequate funding, insufficient experiential training, delay in recruitment of newly trained essential staff and provider disinterest in profession.

**Conclusion:**

Setbacks in the training and recruitment systems in Ghana, inadequate investment in rural health coupled with extent of health provider inherent disposition to practice may be partly responsible for sub-standard obstetric care in the study area. Interventions targeting the afore-mentioned areas may reduce events of sub-standard care.

**Supplementary Information:**

The online version contains supplementary material available at 10.1186/s12913-024-10936-x.

## Abstract (significance)

**What is already known on this subject?:**

Quality of emergency obstetric and newborn care is sub-standard in sub-Saharan Africa.

**What this study adds?:**

Sub-standard emergency obstetric and newborn care in rural Ghana may be due to hitches in placement, training and recruitment processes, centralisation of EmONC, inadequate funding, insufficient experiential training, delay in posting of newly trained essential staff and provider disinterest in profession. Adequate and timely resourcing of health facilities and standardisation of admission procedures into schools may reduce events of sub-standard care.

**Supplementary Information:**

The online version contains supplementary material available at 10.1186/s12913-024-10936-x.

## Background

In recent years, maternal and neonatal mortality rates have witnessed downward trends globally due to several maternal interventions. The rate of decline is, however, slow in sub-Saharan Africa making it unlikely to achieve SDG 3 which targets 70 deaths per 100,000 live births by 2030 and a neonatal mortality rate of less than 12 per 1,000 live births [1]. EmONC is one of the safe motherhood strategies that are aimed at improving maternal and neonatal outcomes [2]. The strategy is an attempt to addresss challenges associated with access to quality emergency obstetric care by empowering selected health facilities with essential drugs, equipment and personnel to be able to perform life saving basic and comprehensive interventions to avert preventable mortalities during childbirth [3]. EmONC operates at two levels: basic and comprehensive. Basic Emergency Obstetric and Newborn Care (BEmONC) facilities perform signal functions such as administration of parenteral antibiotics, oxytocic drugs and anticonvulsants, manual removal of placenta, removal of retained products (manual vacuum extraction, dilatation and curettage), assisted vaginal delivery such as vacuum extraction and foceps delivery and perform basic neonatal resuscitation using bag and mask. Comprehensive Emergency Obstetric and Newborn Care facilities (CEmONC) perform all these in addition to providing Cesarean section (CS) and blood transfusion. It is reckoned that the expansion of EmONC services to rural and disconnected communities will create the opportunity to provide sufficient life-saving services as well as enhance access to good quality care to parturient women.

Studies evaluating quality of EmONC have often focused on how the availability of resources influence quality maternal-neonatal outcomes [4–6]. The results portray inadequate resources for EmONC delivery leading to some adverse maternal and neonatal outcomes in countries across sub-Saharan Africa [7]. Similarly, studies in Ghana record concerns such as inadequate clinical knowledge and competences of health providers and lack of essential supplies as factors constraining quality EmONC provision [8–10]. Investigating into why these issues persist in the quality of maternal and child health care literature space, Filby and colleagues [11] in a systematic review, discovered lack of investiment in midwifery education, inadequate numbers of staff, lack of affordable transport, weak facility management and poor working conditions as main influencers. Relatedly, Geleto, Chojenta, Taddle and Loxton [12] revealed a lack of treatment protocols, poor supportive supervision and poor staff motivation as tailbacks to the provision of quality obstetric care in hospitals in Ethiopia. These studies did not only peruse the views of only health providers, they concentrated on professional factors and failed to uncover issues pertaining to other key components of EmONC notably the role of drugs and equipment. Understanding a Ghanaian situation from a stakeholder perspective may reveal additional nuances that may lead to adoption of appropriate strategies to improve upon maternal and newborn care especially in rural Ghana. The objective of the study is to explore stakeholder opinions on factors triggering sub-standard EmONC delivery in Northern Ghana.

## Methods

### Study design

A qualitative study design from a constructivist paradigm was used for the study [13]. The design enabled the researchers to reflect on stakeholder perspectives and opinions as staunchly as possible while admitting their own reflexive influence in the interpretation of data [14].

### Study setting

Ghana is a country with a striving health care system. The health system has the onerous task of meeting the health needs of the rapidly growing population of Ghana and fighting illnesses associated with poverty and lack of education [15]. Although life expectancy has improved with declining rate of maternal and neonatal mortalities, the system is saddled with challenges such as poor coverage, poor quality of health care, corruption and weak management [16]. These challenges are very much prevalent in the Northern Region where this study was conducted. The region is largely rural, deprived and had a maternal mortality ratio of 207 per 100,000 live births, higher than the national average as at the end of 2017 [17]. At the time of the study, the region had at least one designated BEmONC facility in each rural district as a first level facility and a total of three CEmONC facilities sited in some towns [18]. Two each of BEmONC and CEmONC facilities were selected from these existing facilities for the study. The selected CEmONC facilities had an average of 250 bed capacity [19]. The study population was purposively recruited from these facilities. They comprised 42 EmONC service providers, who were mostly midwives, seven management members, 15 clients who received EmONC services and five care takers. Aside providers’ willingness to partake in the study, the sampling procedure considered years of service, role and gender. Such people were in a unique position to provide relevant information pertaining to the quality of EmONC. Clients comprised women who received EmONC and their care takers. They were selected at the facilities during care delivery and sampling progressed till data saturation.

### Ethical statement

Ethical approval for the study was obtained from the Ghana Health Service (GHS-ERC004/04/19) and the University of Cape Coast [UCC] Ethical Review Board (UCCIRB/CES/2019/03). All methods were carried out in accordance with relevant guidelines and regulations stipulated by the ethics committees.

### Data collection instruments

Both interview and focus group discussion guides were developed based on the study purpose and in conformity with qualitative research methodology [20]. Table s1 contains sample questions on the instruments. The questions sought to explore individual and group views on sub-standard care in the region. They explored issues pertaining to EmONC’s key components namely availability and quality of emergency obstetric drugs, equipment and essential personnel and how and why these contributed to sub-standard care. Probes and prompts were used to elicit detailed submissions from participants. Questions for both IDI and FGDs were pre-tested and corrections made before final administration.

### Data collection

Interviews with management members were conducted in English and lasted an average of 62 mins. Interviews with clients took place upon discharge from facilities and at places suitable to them. A field assistant interpreted the instrument from English into the local language (Dagbani) to elicit views of clients on the subject matter. Consent of participation was obtained using a consent form which required participants to append their signatures or thumbprints as an indication of voluntary participation. Participants were assured of confidentiality and anonymity of information provided through the use of pseudonyms. Six FGDs were held with midwives and anesthetists working at the facilities to explore group views on root causes of sub-standard care. The number of focus groups was determined using Guest and colleagues recommendation on data saturation [21]. FGDs were moderated by the first author who ensured each participant had a fair chance of participation. Both interviews and FGDs were audio taped using an audio device with consent from participants. A total of 9 health providers and 6 clients declined to participate in the study for personal reasons.

### Data analysis

Data were analysed using Braun and Clarke’s [22] thematic analysis procedure. The six staged iterative analysis procedure comprised transcription, reading and familiarisation, coding, searching for themes, reviewing themes, defining and naming themes and finalising the analysis. Audio files were first transcribed into texts by the first author. The transcribed documents were checked for accuracy by the second and third authors. They were then read meticulously for content familiarity and thematic coverage. It was followed by slow reading accompanied by a preliminary assignment of codes. Adhering to a pattern of occurrence process, similar codes were brought together to form sub-themes and then combined to form candidate themes across transcripts. The candidate themes were reviewed in relation to the available data by co-authors and changes made to some codes eliciting a renaming of some themes. Triangulation was performed by identifying the commonality of codes within and across transcripts. The codes were captured into a refined list of themes and sub-themes which were used in promulgating the study findings. Member checking was carried out by contacting some randomly selected participants and gaining feedback on the data interpretations and conclusions.

## Results

The findings demonstrate that sub-standard EmONC delivery is a product of systemic and personal challenges in the region.

### Participant characteristics

A specialist gynaecologist, two unit heads, two hospital matrons, two hospital administrators (referred to as management members), forty midwives and two anesthetists were the health care providers who participated in the study as well as 15 clients and five care takers. These were selected out of a total of two specialist gynaecologists, 10 unit heads, two matrons, eight administrators, 69 midwives and seven anesthetists who made up the total of the provider population at the study facilities as well as a minimum of 78 women who visited the facilities monthly to receive EmONC [23]. Key management members involved in the study had acquired at least a tertiary level education in their respective fields and practised for an average of 27 years. Fourteen of the midwives possessed an earlier Post-secondary certificate indicating they were either Community or Enrolled nurses who went on to specialise in Midwifery. The rest of the midwives were either diploma or degree holders. Most of them had practised for an average of ten years. Clients who were treated of complicated deliveries were mostly of middle or advanced age, less educated hence engaged in petty trading and in polygynous marriages. Care takers possessed similar background characteristics. The most commonly reported obstetric complication was post-partum haemorrhage.

Four themes and 13 sub-themes emerged as root drivers to sub-standard care. Themes identified comprised centralisation of EmONC, inadequate commitment, training and placement issues and providers’ intrinsic factors. These are presented with corresponding sub-themes.

### Theme: Centralisation of EmONC

#### Non-functioning BEmONC facilities

Participants disclosed that though BEmONC facilities earlier received essential equipment to perform signal functions, they were debarred by the country’s National Health Insurance Authority (NHIA), from doing so citing capacity reasons. Such facilities were rather required to conduct routine maternity care while referring all complications to the comprehensive level facilities that had the expertise to handle such cases. As such, they were denied emergency medications needed to treat basic complications. According to them, such an approach promoted needless referrals that imposed undue burden on providers at the comprehensive level facilities, increasing their workload and leading to adverse outcomes in some instances.

We have often been told by Health Insurance officers to escape risk….so I am referring all the cases and burdening my colleagues at that end while I am sitting, is it right? We can manage complications such as post-partum haemorrhage and pre-eclampsia if they give the drugs but they say no, refer. (FGD 4-midwife-BEmONC facility).

So if a woman goes through normal vaginal delivery and is bleeding afterwards, I should not give any medication, I should send her to the hospital… What if she doesn’t survive, what happens and trust me, some of them truly die through that or lose their babies” (FGD 1- midwife-BEmONC facility).

### Problems with referrals

Participants lamented that the poor road network system in the region served as a barrier to access health services in a timely mannner. They maintained that most settlements within the study area are hard to reach as they are cut off by rivers and streams and bumpy dusty roads. This made access to health care difficult necessitating home deliveries. Mothers who encountered complications due to home delivery and their newly born had little chance of receiving the needed intervention at the nearest EmONC health centre as it lacked the resources to intervene and to facilitate referral. A provider explained:

“...Sometimes, the midwives will want to refer the patient but no means of transport to go”.. (FGD 4-midwife-BEmONC facility).

Providers further revealed that due to problems associated with referrals, women and neonates in critical conditions did not survive.

They bring some women who deliver at home and are bleeding maybe due to retained placenta. When they arrive, we will have to refer instead of providing the needed care and the referral process may take time hence causing a delay. (FGD 1-midwife-BEmONC facility).

### Clients’ reactions to referral

Evidence also showed that some clients and care takers resisted referrals to the CEmONC facilities located in the cities because it imposed transportation and associated costs on them. Hence they returned to their homes to try alternative care leading to deaths in some instances.

When we even refer, they don’t want to go. They will be begging that we should treat them at the health centre and some just take their folders and return to their homes and the next moment, we hear the client is dead together with the baby. (FGD 4 midwife-BEmONC facility).

The assertion was confirmed by a care taker:

When we came, the midwife told us to go to the hospital in the city because she didn’t have the means to treat us here. But we didn’t have money to go to the city so we returned home. (Care taker 16).

### Theme: Inadequate commitment

#### Delayed / scant funding

Although CEmONC facilities were mandated to provide both BEmONC and CEmONC signal functions, the facilities studied did not receive adequate financial resources to facilitate care delivery. Some management members disclosed that the hospitals sometimes lacked some basic drugs due to the NHIS inability to promptly reimburse them. Hence, they sometimes procured medicines from the open market on credit and clients had to purchase these on out-of -pocket terms at the hospital pharmacy. Some clients, however, were unable to pay for drugs used due to poverty leading to an eventual depletion of drugs in the pharmacy and a great difficulty in managing complications.

We try to buy the drugs on hire purchase to enable us provide care so when clients come, we write it for them and they send it inside the pharmacy and buy there…that is better than the open market but the people come and they can’t even afford the small token we ask them to pay, yet we have to treat them, so we end up not having drugs in the pharmacy. (1DI-Matron 1-CEmONC facility).

A client disclosed:

“...During the surgery, we had to buy some drugs from the stores in town because the pharmacy didn’t have drugs...” (Client 9).

The scarcity of emergency obstetric drugs led to administration of less effective drugs on some occasions. Participants in a focus group disclosed that some drugs bought from drug stores on the market were found to be fake. Nonetheless, these were used in managing cases. They lamented that the Northern part of the country, in general, was not well served in terms of equitable distribution of health resources.

“...Tell me how many pharmacies in the nation’s capital will you see fake drugs? It’s all over the Northern region here...”(FGD 1-CEmONC facility)

“...They last supplied us with magnesium sulphate but it was expired...” (FGD 3-midwife-BEmONC).

#### Poor maintenance culture

Participants further ascribed the current state of care delivery to poor maintenance culture. They admitted that though essential equipment were occasionally supplied to the health facilities, they went missing the next moment while others got damaged due to malhandling. As a result, providers depended on their clinical judgement in most cases to proceed with care delivery which did not reflect best standard practice. The following quotes express the issue:

If you go to the in-charges’ offices right now, you will see the packs that contained the equipment they supplied us but you get to the wards and they are not there. So if we can’t get the BP readings, for instance, we base our judgement on strength of urine protein to guess pre-eclampsia and treat as such. It might not be the best approach but at least, we are making an effort. (FGD 3-midwife-CEmONC facility).

We supply the units with the necessary equipment periodically but you go round later and they are either broken down or missing and they can’t report because they will have no justification so they struggle like that. (IDI- Matron 1-CEmONC facility)

#### State property mentality

The concept of “public property” reflects irresponsible posture assumed by some public sector workers in their interaction with state property. Some participants hinted that the possible feeling that staff did not own the items they worked with coupled with a weak monitoring system influenced how equipement were handled and how long they lasted leading to “no equipment” in some instances.

To be honest, some of us are reluctant taking care of these things because of the feeling that it isn’t our personal property and the in-charges are not doing much to ensure that the right number of equipment are handed over from shift to shift so we end up being left with nothing to work it. (FGD 2- midwife CEmONC facility)

### Theme: Training and placement issues

#### Non-standardised admission procedures into health training schools

Participants revealed that though quality of EmONC has improved over the years, it could have been better. They blamed the lapses on training and placement of health staff. They indicated that admission into health training schools in the country has become very competitive in recent years. This has led to the introduction of a “protocol list”. It is a list purported to emanate from influential persons (mostly political office holders) in the Ghanaian society whose request for admission for some persons or relatives must be granted regardless of their performance at admission interviews. The evidence suggests that such an approach interferred with the quality of trainees enlisted for training giving rise to poor work ethics and output in health facilities.

At first, when admission was on merit basis, it was better. Now the protocol midwives are too many and they don’t co-operate because when they are doing the wrong things and the other midwife complains, they fight them and as the in-charge you can’t do anything to them because they can even call the big men and report you and you may be removed from your post. (IDI- Unit head 2- CEmONC facility).

#### Inadequate experiential training

Participants in managerial positions further blamed the low skill level of newly trained staff on the educational system in the country. They alluded that the curriculum for instruction especially in health training schools centered more on theory than practice. They further disclosed that, the contact hours for clinical training was inadequate and a subsequent one year after school training in a health facility did not do much in improving the clinical competence of newly trained staff.

The problem is about the quality of the schools and it is at all levels across the country. They are getting inexperienced people who do not have adequate knowledge on what they teach and the teaching materials are also not there to facilitate the work… For clinical students, it was suggested at a meeting that clinical rotations should be two years instead of one. (IDI- Specialist CEmONC facility).

The assertion was corroborated in a focus group discussion:

“...We normally do not understand most things taught so we just memorise to pass…….we don’t also get enough clinical experience while in school so it is on the job that we actually learn...” (FGD 1- midwife- CEmONC facility).

#### Delay in recruitment of newly trained staff

The technical inadequacy especially among newly recruited staff was further traced to the delay in their recruitment. Some newly posted midwives alleged that they had to stay home for two to three years before they were employed due to the government’s inability to immediately absorb them upon completion. As such, they tend to forget most learnt procedures due to a prolonged stay at home and were unable to perform some required tasks when they were eventually employed.

Just imagine that a woman with a bleeding case was brought to the ward. The midwife should have checked her vitals before deciding on what to do but she didn’t. She transfused her with ringer’s lactate before proceeding to check for the cause of bleeding not knowing the client had high blood pressure and then she started fitting. The midwife should have given normal saline but according to her she had forgotten the right procedure. (IDI- Matron 2- CEmONC facility).

#### Refusal of postings to Northern Ghana

Newly posted midwives disclosed that most of their colleagues who were posted to the Northern part of the country refused postings but rather negotiated their way to practice in facilities in the Southern sector. They mentioned the lack of social life and other incentives in Northern Ghana as reasons for their refusal. This perennial attitude creates a serious shortage of midwives in Northern Ghana thereby imposing a daunting effect on the few workers and the care provided.

Some of my friends refused to come here. They said the place is not developed and the weather too is harsh; It is mostly hot, dry and windy due to its proximity to the Sahara desert. (FGD 4- midwife- CEmONC facility).

In an in-depth interview, a manager indicated:

They post newly trained midwives here but few come and they even go on transfer after a short stay. So we always have to battle with shortage of staff. (IDI- Matron 1- CEmONC facility).

### Theme: Intrinsic factors

#### Disinterest in profession

Evidence further suggests that aside from persons who gained admission into schools through favours, others pursued midwifery or related programmes due to persuasion from family members. A newly recruited midwife disclosed that she studied midwifery based on the advice of her father. Relatedly, some category of nurses who had earlier attained certificates to provide basic preventive care were motivated to pursue midwifery as a form of career advancement and a bid to improve the number of midwives in the country. Some other management members disclosed that the job readiness nature of the profession motivated some young girls to enroll in it and not the passion for the work and this influenced how they worked.

Actually, me, I didn’t like midwifery. My father advised me to do it. He said it is better than general nursing. (FGD 5- midwife- CEmONC facility).

For some of these young midwives of today, their stay in Midwifery is but a missed call. They are here because they need a job and not for service. (IDI-Matron 2-CEmONC facility).

“...They said those of us with certificate background can only be granted study leave to pursue midwifery so we had to do it for promotion sake...” (FGD 1-midwife-CEmONC facility).

#### Haemophobic and emetophobic health staff

It was also revealed that some practitioners abhorred contact with blood while others could not withstand a vomiting scene from labouring women. Some obstetric nurses (male midwives) doubled as teachers of the Quran (mallam) and would not taint themselves with unclean blood from labouring women. In their shift, cases had to wait for other midwives to attend to and the delays heightened client’s risk. Though management members disclosed that staff of this calibre were released from the facilities because of non-performance, such people possibly continued to pose as threat to maternal and neonatal health at their next post.

A participant shared the following in terms of males in midwifery:

You see some of these male midwives, the zeal is not in them. The females are even better. Some say they are Mallams so they won’t touch blood. If so, why did they decide to do midwifery? When they are on duty, a lot of cases go bad so I have to always pair them with the serious ones. (IDI- Unit head 1-CEmONC facility).

In a FGD, the following emerged:

As I speak with you, two of my colleague male midwives were released in the same month and they left the hospital because though they came to work every day, they never attended to clients. They either lazed about or used their phones throughout their duty period meanwhile clients will be in need of care. (FGD 4-midweife- CEmONC facility).

They are some of them who run away at the sight of vomit. How can a midwife fear vomit? (Matron 1- CEmONC facility).

#### General apathy with associated attrition

Lack of passion, especially in midwifery, led to series of apathetic behaviours generally. Participants asserted that some colleagues deliberately refused to attend periodic in-service training aimed at skill improvement at the facilities due to apathy. This made them incapable of performing certain clinical tasks. Some others also left the field after few years of practice to persue other programmes of interest thereby creating a brain drain. Unfortunately, there was no immediate replacement in the vacancy created and this worsened the already precarious shortage of midwives in the area affecting the quality of obstetric care.

Three of my midwives left for school last year. They said they want to pursue Public Health instead. (IDI- Specialist).

Another provider lamented:

Sometimes, you can’t help but to wonder why there are so many professions and yet A or B is here, why are they here? How can someone say they cannot perform certain signal functions when they do organise such workshops here? (FGD 5- midwife- CEmONC facility).

However, staff who maintained they had passion for their career, endeavoured to give off their best at work.

I think that passion for my job is the right expression because I love what I do. No matter how tired I am if I am on duty, you see that I am filled with so much energy that I don’t know where it even comes from so I really have passion for my work. (FGD 2-midwife- CEmONC facility).

Managers affirmed that some young health workers were good and devoted but such people were rather few and had less influence on the nature of care that was provided.

“...We have just a few serious and committed ones...” (IDI- Administrator 2- CEmONC facility).

A client also indicated:

As for the young midwives, most of them don’t respect us, just a few. Maybe it is because they haven’t given birth before to experience how painful it is.(Client 12).

## Discussion

Good quality EmONC is crucial to reducing maternal and neonatal morbidity and mortality [24]. Regrettably, the state of EmONC in sub-Saharan Africa has often been described as sub-standard [25]. This study sought to establish the underlying nuances accountable for this situation in rural Ghana by exploring the opinions of key stakeholders. The findings highlight centralisation of EmONC, inadequate commitment, hitches in training and placement processes and provider intrinsic factors as main drivers to sub-standard care.

Centralisation of EmONC emerged as a useful factor to the course of inadequate care reported in the current study. This finding confirms many others that have cited referral-related challenges as instrumental to most adverse maternal and neonatal outcomes [26, 27]. Evidence from this study suggests that the situation in Ghana and similar sub-Saharan African countries might be reduced through prioritisation of rural health [28]. In Ghana, health facilities are categorised into primary, secondary and tertiary to differentiate between the complexities of medical cases and to enable referral of cases to higher levels for continuous care. While this arrangement might be expedient in promoting quality, it might not be suitable for rural areas where access to care is a challenge due to geographical factors [9]. Therefore, the current emphasis on centralising EmONC in regional and some referral hospitals in urban centres, at the neglect of BEmONC facilities, may not serve the needs of rural women who need these services most [29]. It is thus crucial for governments to place priority on rural health by empowering BEmONC facilities in these areas with essential drugs, supplies and experienced staff. This, together with effective supervision and monitoring, may facilitate effective BEmONC delivery and curtail needless referrals.

Ghana’s quest to reducing its maternal mortality targetted infrastructural improvement and an increase in human resource base through added training [30]. However, this study established that the current education and training system is deficient in producing the right middle level workforce for the country’s needs. This is similar to previous studies [31, 32]. According to the International Confederation of Midwives (ICM), midwives should be able to demonstrate competency in the provision of care during pregnancy, labour, birth and the post natal period [33]. The inadequacy of skills in these areas has partly been linked to inadequacies in education and training as well as socio-political influences. Principally, the curriculum for instruction in Ghana is generally theory driven [34] due to lack of human and material resources to adopt much more practical approaches. Although clinical staff are further expected to gain experience through in-service training, this provision seems insufficient to equip beginners with the clinical skills required to transition to practice. The situation is exacerbated by delay in posting of newly trained staff as government is unable to immediately absorb trainees after school due to fiscal policy and budgeting reasons. Ayawine and Atinga [35] reported elsewhere that delay in recruitment of staff led to harzadous care delivery as some staff adopted trial and error management procedures on clients in order to revamp lost skills. Ederer et al. [36] also attest that such service providers are a threat to patients’ safety as they are more likely to commit avoidable errors which endanger lives. To mitigate against this trend, the curriculum for health training schools may have to be rooted in emerging pedagogy that meets national and international standards in terms of knowledge acquisition and application. Students may need to spend adequate time in clinical settings where real life situations exist in order to garner the skills required for effective practice. They may also be placed under a temporary mentoring scheme or a tranisiton to practice program after training, to undergo on the job training from experienced staff as a means of improving upon their skills before they are formally employed.

Provider disinterest also emerged as a reason for sub-standard care in this study. While studies in developing countries report external factors such as poor working conditions and low opportunity for career advancement as distracts to good quality care [37, 38], this study identified provider internal motivation as a major forerunner to sub-standard care. It is argued that health staff who are intrinsically motivated tend to give off their best at work. In a study among 12 European countries, Aiken and colleagues [39], highlighted that a substantial proportion of nursing professionals disclosed their dissatisfaction with their career and intended to vacate post the subsequent year. Nonetheless, in Turkey, albeit some seeming constraints, midwives derived inner satisfaction helping and being with women and did not wish to abandon their profession in the probable future [40]. The authors thus conceded that intrinsic motivation has a more positive impact on quality of care and general productivity. Maloney et al. [41] also revealed that motivated staff may overcome some barriers to improve quality of care while disinterest providers will exhibit reluctance to learn new things and will not be productive. Similarly, while some staff in this study disclosed they were passionate about their job, others were haemophibic or emetophobic and this affected practice. Another group maintained they were coerced by relatives to pursue certain programmes and did not have personal interest and passion for the work. It is established that these calibre of workers may not only be inefficient and irresponsible at work, they are more likely to complain of burnout, dissatisfaction, or change job thereby constantly creating a service gap for health professionals [41]. It is important to place priority on personal background of prospective candidates as part of the admission processes into health training institutions. This may be achieved by denouncing a “protocol list” and including a clinical psychologist as part of the admission team to ascertain students’ innate ability and interest through appropriate clinical simulation exercises. This approach may help identify and train personnel with the right disposition to practice.

## Conclusion

Setbacks in the training and recruitment systems in Ghana, inadequate investment in rural health coupled with extent of health provider inherent disposition to practice may be partly responsible for sub-standard obstetric care in the study area. Providing adequate resources for BEmONC facilities, adhering to a standardised admission and screening procedure into health training schools and curriculum modification in pedagogy may motivate and enhance the competence of newly trained essential workforce and guarantee better health outcomes among rural mothers.

## Implications for practice

Inadequate funding of EmONC facilities induces poor quality care that poses a threat to maternal and neonatal health especially in rural Ghana. There is the need to adequately resource rural facilities with experienced staff and essential logistics to facilitate access to critical care while enabling CEmONC facilties with similar logistics to be able to perform core signal functions. Curriculum modification may also enhance the competence of newly trained essential staff. Ghana and other developing countries may need to re-think their strategies of improving upon maternal health by not concentrating mainly on massive production of essential staff but by raising qualified and passionate workforce using objective and standardised means and absorbing them immediately after training to promote effective care delivery.

## Strengths of the study

A major strength of the study lies in the use of multiple stakeholders and data collection methods to unravel the intricacies underlying the phenomenon under study. This approach enhances the trustworthiness of the findings. The study also sdopted an analytical framework in the presentation of results through the use of thick descriptions and concrete details of events in the study environment. Such a style allows for duplication in comparable settings.

## Limitations

Exclusion of officials from the National Health Insurance Scheme (NHIS), being a major stakeholder in health care delivery in Ghana, might have led to loss of data that could have strengthened the findings of the study.

### Electronic supplementary material

Below is the link to the electronic supplementary material.

Supplementary Material 1

Supplementary Material 2

Supplementary Material 3

Supplementary Material 4

Supplementary Material 5

## Data Availability

The datasets used and/or analysed during the current study are available from the corresponding author on reasonable request.

## Abbreviations

BEmONC: Basic Emergency Obstetric and Newborn Care
CEmONC: Comprhensive Emergency Obstetric and Newborn Care
EmONC: Emergency Obstetric and Newborn Care
FGD: Focus group discussion
GHS: Ghana Health Service
ICM: International Conferedration of Midwives
IDI: In-depth Interview
NHIA: National Health Insurance Authority
NHIS: National Health Insurance Scheme
SDG: Sustainable development goal
UCC: Univeristy of Cape Coast
