# Supplementary material for: Stakeholder opinions on perceived sub-standard emergency obstetric and newborn care in Ghana
Source: BMC Health Serv Res. 2024 Apr 12;24:461. doi: 10.1186/s12913-024-10936-x (PMC11015552; doi:10.1186/s12913-024-10936-x)
Supplement: Supplementary file 1 — Supplementary Material 1 [file 12913_2024_10936_MOESM1_ESM.docx]

**Table.s1: Sample questions**

|  | Interview questions  **Management members** | Interview questions  **Clients / care takers** | Focus guide  **Health care providers** |
| --- | --- | --- | --- |
| Socio-demography  Participant number………  Age………….  Educational background…….  Cadre of staff……….  Number of years in practice…….  Clients  Participant number………  Age………….  Educational  Occupation….  Marital status…  Type of obstetric complication… | -Are you able to perform the BEmONC/ CEmONC signal functions as required of your facility? How / why?  -What is your opinion about the content of care provided?  *-NB: Probe in terms of drugs, equipment and essential personnel*  -What may be responsible for the nature of care provided? | -Were you or your relative able to access emergency obstetric care during or following delivery? How /Why?  -What is your opinion about the content of care provided?  *-NB: Probe in terms of drugs, equipment and essential personnel*  -What may be responsible for the nature of care provided | -Are you able to perform the BEmONC/ CEmONC signal functions as required of your facility? How / why?  -What is your opinion about the content of care provided?  *-NB: Probe in terms of drugs, equipment and essential personnel*  -What may be responsible for the nature of care provided |
