# Supplementary material for: Stakeholder opinions on perceived sub-standard emergency obstetric and newborn care in Ghana
Source: BMC Health Serv Res. 2024 Apr 12;24:461. doi: 10.1186/s12913-024-10936-x (PMC11015552; doi:10.1186/s12913-024-10936-x)
Supplement: Supplementary file 2 — Supplementary Material 2 [file 12913_2024_10936_MOESM2_ESM.docx]

**Table.s2: Participants’ details for key management members**

| **Variable** | **Number of participants** |
| --- | --- |
| **Sex**  Female  Male | 4  3 |
| **Age**  30-39  40-49  50+ | 3  2  2 |
| **Education**  Post-secondary  Tertiary | 2  5 |
| **Rank**  Principal midwifery officer  Deputy director of nursing services  Obstetrics and gynaecology specialist  Senior administrator  Principal administrator | 2  2  1  1  1 |
| **Years in practice**  1-10  11-20  21-30  31-40 | 1  1  2  3 |
| Total number of participants | 7 |
