# Supplementary material for: Stakeholder opinions on perceived sub-standard emergency obstetric and newborn care in Ghana
Source: BMC Health Serv Res. 2024 Apr 12;24:461. doi: 10.1186/s12913-024-10936-x (PMC11015552; doi:10.1186/s12913-024-10936-x)
Supplement: Supplementary file 3 — Supplementary Material 3 [file 12913_2024_10936_MOESM3_ESM.docx]

**Table.s3: Participants’ details for health providers**

| **Variable** | **Number of participants** |
| --- | --- |
| **Sex**  Female  Male | 40  2 |
| **Age**  20-29  30-39  40-49 | 6  32  4 |
| **Education**  Post-secondary  Tertiary | 14  28 |
| **Rank**  Staff midwife  Senior staff midwife  Senior midwifery officer  Senior anesthetists | 6  20  14  2 |
| **Years in practice**  1-10  11-20  Total number of participants | 26  16  42 |
|  |  |
