# Supplementary material for: Stakeholder opinions on perceived sub-standard emergency obstetric and newborn care in Ghana
Source: BMC Health Serv Res. 2024 Apr 12;24:461. doi: 10.1186/s12913-024-10936-x (PMC11015552; doi:10.1186/s12913-024-10936-x)
Supplement: Supplementary file 4 — Supplementary Material 4 [file 12913_2024_10936_MOESM4_ESM.docx]

**Table.s4: Participants’ details for clients**

| **Age*** | **No of Participants** |
| --- | --- |
| Below 20 | 2 |
| 20-29 | 7 |
| 30-39 | 4 |
| 40-49 | 7 |
| **Education*** |  |
| None | 13 |
| Basic | 5 |
| Secondary | 1 |
| Tertiary | 1 |
| **Occupation*** |  |
| Unemployed | 5 |
| Informal | 14 |
| Formal | 1 |
| **Marital Status*** |  |
| None | 1 |
| Monogamy | 4 |
| Polygyny | 15 |
| **Type of obstetric complication obtained** |  |
| Post-partum haemorrhage | 9 |
| Pre-eclampsia | 4 |
| Placenta praevia | 1 |
| Maternal distress | 1 |
| **Total number of clients and care takers** | 20 |

***** Numbers reflect both clients and care takers
