# Supplementary material for: Stakeholder opinions on perceived sub-standard emergency obstetric and newborn care in Ghana
Source: BMC Health Serv Res. 2024 Apr 12;24:461. doi: 10.1186/s12913-024-10936-x (PMC11015552; doi:10.1186/s12913-024-10936-x)
Supplement: Supplementary file 5 — Supplementary Material 5 [file 12913_2024_10936_MOESM5_ESM.docx]

**Table.s5: Summary of study findings**

| **Theme** | **Sub theme** |
| --- | --- |
| Centralisation of EmONC | -Non-functioning BEmONC facilities |
|  | -Problems with referrals |
|  | - Clients’ reactions to referrals |
|  |  |
| Inadequate commitment | -Delayed / scant funding |
|  | -Poor maintenance culture |
|  | -State property mentality |
|  |  |
| Training and Placement Challenges | -Non-standardised admission procedures into health training schools |
|  | -Inadequate experiential training |
|  | **-**Delay in posting of newly trained staff |
|  | - Refusal of postings to Northern Ghana |
|  |  |
| Intrinsic Factors | -Disinterest in profession |
|  | -Haemophobic / emetophobic staff |
|  | -General apathy and attrition |
|  |  |
